# Resilience, Cardiological Outcome, and Their Correlations With Anxious-Depressive Symptoms and Quality of Life in Patients With an Implantable Cardioverter Defibrillator

**DOI:** 10.3389/fpsyt.2021.763726

**Published:** 2021-11-24

**Authors:** Celeste Isella, Alessandra Gasparini, Giulia Lucca, Marta Ielmini, Ivano Caselli, Nicola Poloni, Carlo Dajelli Ermolli, Fabrizio Caravati, Battistina Castiglioni, Roberto De Ponti, Camilla Callegari

**Affiliations:** ^1^Department of Medicine and Surgery, Section of Psychiatry, University of Insubria, Varese, Italy; ^2^Department of Heart and Vessels, Ospedale di Circolo, University of Insubria, Varese, Italy

**Keywords:** implantable cardioverter defibrillator, resilience, anxiety, depression, life quality, screening, outcome

## Abstract

**Background:** Resilience is proven as a protective factor against the development of psychiatric disorders, and it has gained clinical relevance in the development and progression of cardiovascular pathology. The authors performed a longitudinal study on patients with implantable cardioverter defibrillator (ICD) with the primary aim to highlight the possible existence of a correlation between individual resilience capacity, depressive and anxiety symptoms, and quality of life in terms of outcomes. The secondary aim was to analyze the differences between patients with major cardiac events in the follow-up and patients without cardiac events with respect to the previous variables.

**Materials and Methods:** A total of 80 patients enrolled in the Cardiology Unit were evaluated at T_0_ and during the follow-up through the following scales: the *14-item Resilience Scale* (RS-14), the *Hospital Anxiety and Depression Scale* (HADS), and the *World Health Organization Quality of Life-Brief Version* (WHOQOL-Bref).

**Results:** A significant linear correlation between resilience and all the areas of quality of life at T_0_, T_1_, and T_2_ emerged. A negative correlation between resilience and anxiety and depressive symptoms emerged, as well as between depression and anxiety and quality of life. Patients with cardiac events during the follow up have shown a worse quality of life and the onset of anxiety-depressive symptoms over time, without changes to the resilience scores. Patients without cardiac events showed an increasing trend in resilience scores.

**Discussion:** Given the speed and simplicity of use of the RS-14 scale, it seems promising to further investigate the real clinical usefulness of this instrument in the cardiology field.

## Background

In recent years, resilience, defined as the “universal capacity which allows a person, group or community to prevent, minimize or overcome the damaging effects of adversity” (1), has gained clinical relevance in several medical disciplines, with particular attention to development and progression of chronic diseases. In fact, resilience not only moderates the link between risk factors and outcome but also allows maximizing the benefits coming from specific interventions, aimed at recovering from specific pathologies (2, 3). Indeed, resilience has proved itself effective in allowing a more rapid remission, increasing coping skills regarding one's condition and related treatments; this is also in line with current neuroscientific models (4–6). Regarding cardiovascular pathology, resilience is found to be particularly high in patients with coronary artery disease (7), although lower than the general population (8), and is associated with a better outcome (9) and a minor incidence of post-traumatic stress disorder symptoms (10). Some authors (5, 11) found that in cardiovascular population, psychological resilience seems to independently predict patients' mental and physical quality of life but has not been examined in relation to depression and in relation to the use of implantable cardioverter defibrillator (ICD).

Most patients undergoing ICD placement are able, within a span of variable time after the surgery, to recover their normal level of activity (12) with a good degree of satisfaction with the device (13). However, a significant percentage of patients could experience more relevant difficulties, which may persist for a prolonged time. The reported prevalence of common psychological and emotional disorders among carriers of ICD ranges from 15 to 60% (14, 15). The most represented diagnoses are anxiety disorders (24–87%), depressive disorders (9–15%), post-traumatic stress disorder (up to 20%), and adjustment disorders (16, 17).

The link between resilience and depressive symptoms is also well-known; several studies concluded that resilience is a protective factor against the development of psychiatric disorders, such as PTSD and depression (18, 19). Furthermore, negative effects and their reciprocal influences with resilience have been related to an increased risk of mortality in patients with cardiovascular disease (20, 21). In fact, there is an intrinsic relationship between psychological state and arrhythmic risk, demonstrated by the increased incidence of ventricular arrhythmias malignant in subjects with anxiety and depression (11).

Moreover, the psychological sequelae, experienced by ICD patients, may cause an impairment of their quality of life. The perception of a good quality of life in ICD carriers, defined as “*a multidimensional health outcome in which biological, psychological and social functioning are interdependent,”* should be taken into consideration as a success factor for the implant (22).

Given this premise, the authors performed a longitudinal study on 80 patients with ICD with the primary aim to highlight the possible existence of a correlation between individual resilience capacity and depressive and anxiety symptoms, using quality of life in terms of outcomes. The secondary aim was to analyze the differences between patients with major cardiac events (death, hospitalizations, incidence of arrhythmias, and ICD shocks) in the follow-up and patients without cardiac events with respect to the previous variables.

## Materials and Methods

The prospective observational study VC was conducted on 80 patients enrolled in the Cardiology Unit of the “ASST Sette Laghi—Ospedale di Circolo—Fondazione Macchi” of Varese. The study was approved by the Ethical Committee of Insubria on April 10, 2018 (study no. 22). This study observed regulatory and legal requirements (DL n.211, June 24, 2003, and DM December 17, 2004), according to the Declaration of Helsinki's ethical principles. Following the Institutional Review Board approval, all patients were specifically informed about the opportunity to participate in this study and signed a written specific consent before the enrollment. Data collected referred to the period between June 15, 2018 and June 30, 2019. Patients were evaluated at T_0_, at the time of ICD implant, after 3 months (T_1_), and after 12 months (T_2_).

To be enrolled in the present study, patients had to fulfill the following inclusion criteria: patients aged between 18 and 85 years with indication to ICD implant, both in primary and secondary prevention; and willingness to sign the informed consent. Exclusion criteria were life expectancy <12 months, neurodegenerative diseases, moderate or severe cognitive impairment, moderate or severe intellectual disability, depressive and anxiety disorders diagnosis, previous or current psychopharmacology treatment at T_0_, or abuse of alcohol or illicit substances. The following socio-demographic and clinical variables were taken into consideration: gender, age, education, marital status, employment, living conditions, past medical history, length of hospitalization, type of ICD, New York Heart Association (NYHA) (23) class, number of shocks after the implant, hospitalization, and access to the emergency room of cardiological relevance.

The following evaluation scales were administered at T_0_ and during the follow up: the *14-item Resilience Scale*, which allows the assessment of resilience levels according to a two-factor structure (competence personal and self-acceptance) (24–26); the *Hospital Anxiety and Depression Scale* (HADS) that consists of 14 items, 7 of those for anxiety levels, whereas the others estimate depression grades, and that showed acceptable psychometric properties in several medical fields including cardiology (27, 28); and the *World Health Organization Quality of Life-Brief Version* (WHOQOL-Bref), shortened version of WHOQoL-100, which comprises four domains (physical health, psychological health, relationships, and environment) and allows an overall judgment on patients' quality of life and on general health (29, 30).

Collected data were analyzed using SPSS Version 22.0 (IBM Corp., Armonk, NY, 2013). Continuous variables were represented by mean ± deviation standard or median ± standard error in case of non-Gaussian distribution. The qualitative variables were represented by frequencies and distributions of frequency. Once the normal distribution of the scores obtained at the RS-14 was confirmed, comparison of means by ANOVA test was performed; given the non-normality of other data (Lilliefors *p* < 0.05) the Mann-Whitney test was used. The evaluation of the existence of a possible correlation between resilience and other continuous variables collected was made through the calculation of Pearson's R and R2. Authors also performed a multivariate regression considering resilience, depression, and anxiety as independent variables and WHOQOL-Bref scores as dependent variables. The possible existence of a variation of resilience and other psychological variables over time was subsequently assessed, using the ANOVA test for paired data and the Wilcoxon Matched Pairs Test for non-Gaussian data. A *p* value < 0.05 was considered statistically significant.

## Results

Participation in the study was proposed to 80 patients; 75 patients (93.75%) signed the informed consent with subsequent enrollment. At T_1_, 7 patients dropped out of the study because they did not want to be assessed thorough the questionnaires. At T_2_, among the 37 patients recruited for this last phase of study, one patient died not for cardiovascular problems, and 36 completed the follow-up. Socio-demographic and clinical characteristics of samples are listed in Table 1.

**Table 1:** Socio-demographic and clinical characteristics.

	**Total**
**Gender**	
Male	60 (80%)
Female	15 (20%)
**Age**	
Mean	69 ± 10.49
**Education**	
None	1 (1.3%)
Primary school	26 (34.7%)
Junior high school/High school	6 (60%)
Bachelor's degree	3 (4%)
**Marital status**	
Unmarried	4 (5.3%)
Married	58 (77.3%)
Divorced	6 (8%)
Widowed	7 (9.4%)
**Employment**	
Unemployed	14 (28.6%)
Employed	10 (20.4%)
Retired	25 (51%)
**Living condition**	
No caregiver	69 (92%)
Caregiver	6 (8%)
Familiarity for serious mental illness	0
**Past medical history**	
Hypertension	59 (78.67%)
Previous myocardial ischemia	50 (66.67%)
Respiratory pathologies	19 (25.33%)
Neoplastic pathologies	11 (14.67%)
Neurologic pathologies	12 (16%)
Minor psychiatric disorders	4 (5.33%)
**NYHA class T0**	
Class I	40 (53.33%)
Class II	27 (36%)
Class III	7 (9.33%)
Class IV	1 (1.33%)

The hospitalization lasted between 3 and 68 days, with a median of 6 days (95% CI = 8–13 days). Most patients (89.33%) were discharged at home, whereas 8 patients (10.67%) were admitted to a cardiological rehabilitation center after hospital discharge. Implant complications occurred in 5 patients (6.67%): in two cases the cause was arrhythmic, two were on a hemorrhagic basis, and one on an infectious basis. The ICD was implanted in primary prevention in 59 patients (78.67%), while in the remaining 21.33% (*n* = 16) in secondary prevention for sustained ventricular arrhythmias (68.75%; *n* = 11) or cardio-circulatory arrest (31.25%; *n* = 5). Forty patients (53.33%) received a single/dual chamber defibrillator, 40% (*n* = 30) a biventricular defibrillator, whereas the remaining 6.67% (*n* = 5) a subcutaneous device. Table 2 shows changes of the considered psychological variables during the study period (Table 2).

**Table 2:** Psychological variables at T_0_, T_1_ and T_2_.

	**T_**0**_**	**T_**1**_**	**T_**2**_**
RS-14 (mean)	85.52 ± 9.61	88.00 ± 9.40	87.61 ± 5.55
HADS-d (median)	5 ± 0.74	2 ± 0.60	4 ± 0.58
HADS-a (median)	6 ± 0.63	3 ± 0.61	4 ± 0.58
**WHOQoL-Bref (mean)**			
Global	55.25 ± 2.97	50.00 ± 2.76	62.50 ± 2.67
Physical health	66.07 ± 1.40	71.41 ± 1.44	69.65 ± 2.28
Psychological	70.83 ± 1.33	70.83 ± 1.32	70.83 ± 1.41
Social relationship	70.85 ± 1.74	66.67 ± 1.60	66.67 ± 1.70
Environment	65.61 ± 1.90	65.63 ± 1.34	68.75 ± 1.27

By comparing intra-individual's modification of RS-14 score at T0 and during the follow-up, no statistically significant differences emerged (*p* = 0.06).

Regarding the HADS scores, anxious symptomatology improved at the first follow-up after 3 months (*p* = 0.008), though it worsened again after 12 months (*p* = 0.007). On the contrary, depressive symptoms underwent a progressive improvement between T_0_ and T_1_ (*p* = 0.002) then remained stable over time. Regarding the WHOQoL global score, no statistically significant differences were observed between T_0_ and T_1_ (*p* = 0.37), whereas it has improved at the second follow-up after 12 months (*p* = 0.002); no statistically significant differences were observed in the Physical Health and Psychological domains' score over the study period. In the Social Relationship domain, patients' scores worsened at the first follow-up (*p* = 0.027), showing no further variation after 12 months. Lastly, a statistically significant difference was observed in the Environment domain, with a clear improvement after 12 months from the implant (*p* = 0.03).

A significant linear correlation was found between resilience (RS-14) and all areas of quality of life (WHOQoL-Bref), except for the Social Relationship domain, during the whole study period. In greater detail, this correlation appeared stronger for Physical Health and Psychological domains (Table 3). Furthermore, a strong inverse correlation between resilience levels and anxious-depressive symptoms emerged from data analysis; these results remained constant over time during the subsequent follow-up (Table 3).

**Table 3:** Correlation between RS-14 and the other psychological variables.

	**r**	***p*-value**
**WHOQoL-Bref (T0)**		
Global	0.30	0.009
Physical health	0.44	<0.0001
Psychological	0.56	<0.0001
Social relationship	0.34	0.0003
Environment	0.19	0.09
HADS-a	−0.42	0.0004
HADS-d	−0.66	<0.0001

At the first outpatient visit to check ICD functioning (T_1_), it emerged that two patients received a shock from the device; one patient had been appropriately shocked for the onset of sustained ventricular tachycardia, whereas for the other the shock was inappropriate for the onset of atrial fibrillation. By consulting the hospital software, it was also found that 13.24% of patients had one or more accesses to the emergency department or admissions to the cardiology department. In greater detail, 66.67% of the patients had one access only, whereas the remaining 33.33% had two or more accesses. The main causes of hospitalization were arrhythmias (22.22%), heart failure (44.44%), myocardial infarction (11.11%), and other cardiac causes (22.22%).

At T_2_, ICD shocks had been recorded for four patients: two patients received appropriate shocks for the onset of sustained ventricular tachycardia, whereas one patient received three inappropriate ICD shocks for the onset of supraventricular tachycardia. It was also found that 27.03% of patients had made one or more visits to the emergency room or admissions to cardiology department. Leading causes of hospitalization were the presence of arrhythmias (30%) and the onset of heart failure (30%), followed by myocardial infarction (20%) and other cardiac causes (20%).

In the group of patients that concluded the follow-up with events, an improvement has been observed during the whole study period in the Psychological, Social Relationship, and Environment domains, whereas other psychological variables did not show statistically significant variations. No statistically significant differences were found in patients without events (Table 4).

**Table 4:** Changes in psychological variables in patients without and with events (ICD shocks and/or hospitalization).

	**T_**0**_**	**T_**1**_**	**T_**2**_**	**T_**0**_-T_**2**_**	***p*-value**
**Changes in psychological variables in patients without events**					
RS-14 (mean)	84.45 ± 11.17	84.73 ± 10.00	86.18 ± 5.04	*t* = 0.59	0.57
HADS-d (median)	3 ± 1.42	3 ± 1.32	4 ± 0.82	*z* = 0.77	0.44
HADS-a (median)	6 ± 1.15	4 ± 1.26	5 ± 1.30	*z* = 0.12	0.91
**WHOQoL-Bref (mean)**					
Global	50.00 ± 4.85	50.00 ± 5.61	50.00 ± 5.93	*z* = 0.84	0.07
Physical health	60.71 ± 4.03	67.86 ± 3.32	64.29 ± 4.43	*z* = 0.46	0.65
Psychological	58.33 ± 4.93	70.80 ± 2.97	70.83 ± 2.60	*z* = 1.94	0.05
Social relationship	66.70 ± 3.05	66.67 ± 2.41	66.67 ± 2.19	*z* = 2.03	0.04
Environment	59.37 ± 3.07	65.63 ± 2.74	68.75 ± 2.12	*z* = 2.52	0.011
**Changes in psychological variables in patients with events**					
RS-14 (mean)	86.00 ± 9.06	89.44 ± 8.97	88.24 ± 5.75	*t* = 1.29	0.21
HADS-d (median)	5 ± 0.87	3 ± 0.65	4 ± 0.69	*z* = 2.28	0.02
HADS-a (median)	6 ± 0.77	2 ± 0.70	4 ± 0.62	*z* = 1.56	0.12
**WHOQoL-Bref (mean)**					
Global	62.50 ± 3.29	62.50 ± 3.06	62.50 ± 2.79	*z* = 1.38	0.17
Physical health	71.43 ± 2.30	71.43 ± 2.14	71.43 ± 2.61	*z* = 0.69	0.49
Psychological	70.80 ± 2.37	75.00 ± 2.49	70.83 ± 1.73	*z* = 0.89	0.37
Social relationship	75.00 ± 2.16	75.36 ± 2.41	76.12 ± 2.19	*z* = 1.99	0.17
Environment	68.75 ± 2.07	65.63 ± 1.48	68.85 ± 1.53	*z* = 1.38	0.16

Comparing the group of patients, those with events (device shocks and/or hospital admissions for cardiac causes) and those without events, it was found that at time T_1_ the patients with cardiac events presented on average increased levels of anxiety (3 ± 0. 41 vs. 5 ± 1.27; *z* = −1.90; *p* = 0.06) and a significant reduction in the overall quality of life (62.50 ± 2.28 vs. 43.75 ± 6.57; *z* = 2.67; *p* = 0.008). Similar results were found at T_2_: patients with cardiac events had on average a worse overall quality of life (62.50 ± 3.29 vs. 50.00 ± 4.85; *z* = 2.79; *p* = 0.009); furthermore, patients with events show worse scores in the Environmental domain compared to those without cardiac events and/or hospitalization (68.75 ± 2.07 vs. 59.37 ± 3.07; *z* = −2.99; *p* = 0.005). No further statistical differences had been observed in the two groups.

A multivariate regression considering resilience, anxiety, and depression as independent variables and WHOQOL-Bref as the dependent one was performed at each time point of the study.

At T_0_, an association between the physical (*b* = 0.45; *p* = 0.0001), psychological (*b* = 0.47 *p* < 0 0001 areas), and environmental areas (*b* = 0.30; *p* = 0.02) of the WHOQOL-Bref emerged. No independent variables and global and social quality of life were identified. Anxiety was characterized as an independent modifier of the physical area (*b* = −0.28; *p* = 0.02) and depression as an independent modifier of the psychological area (*b* = −0, 26; *p* = 0.03). At T_1_, the multivariate analysis showed that resilience was independently associated with the physical areas (*b* = 0.28; *p* = 0.019), with the social one (*b* = 0.50; *p* = 0.0001), and the psychological one (*b* = 0.48; *p* < 0.0001) of the WHOQOL-Bref. Anxiety (*b* = −0.27; *p* = 0.016) and depression (*b* = −0.28; *p* = 0.04) were also related to the physical and psychological areas (anxiety: *b* = −0.23; *p* = 0.01; depression: *b* = −0.28; *p* = 0.009), while the environmental area showed a correlation only with depression (*b* = −0.40; *p* = 0.022). At T_2_, no significant findings emerged.

## Discussion

Despite the success of cardiac defibrillator implantation for the prevention of sudden cardiac death and in terms of cardiological outcome, there is a growing demand not only to improve the general mortality and safety of patients, but also their quality of life (14). Many patients with ICD experience high degrees of distress in the period immediately before and after the implant, but most of them are usually able to quickly return to normal activities and report feelings of relief. Cardiac events, however, can worsen the individual response to the implant. The purpose of this work was therefore to describe the complex interaction between underlying cardiological disease, ICD implantation, and individual factors of stress vulnerability and resilience on a real clinical sample, in order to better understand the psychological variables on which to intervene in order to obtain a better adaptation and an increase in the quality of life. In fact, emotion regulation has emerged as one of the most important personal competencies in modulating the risk of psychopathological burden (31, 32). Moreover, as in other clinical fields, better health outcomes could also be predicted using the pharmacogenetic information through pharmacogenetic testing as part of routine clinical care in the management of cardiovascular disease (33–35). In general, it is possible to observe that in the year following an ICD implant, patients need a period of adaptation, against which various internal and external factors intervene, with consequences both on a psychological level and on the quality of life. After a year an overall improvement was observed in both aspects; however, for patients who presented problems of a cardiac nature during the observation period, such recovery appears more difficult and slower, also in relation to elements of reinforcement and negative expectations related to body, health, and future. Patients with cardiac events during the follow-up have shown a worse quality of life and the onset of anxiety-depressive symptoms over time, without changes to the resilience scores. Patients without cardiac events showed an increasing trend in resilience scores. Good resilience skills can instead promote faster activation of effective coping strategies, thus allowing faster remission, as confirmed in other works (4). From these results, the need to propose an early screening in order to identify the patients most at risk of developing not only physical but also psychological complications emerges (36, 37). The tests used in the present work have proved to be useful in this regard; in particular, the presence of high levels of resilience, measured through the RS-14, was found to be statistically significantly correlated to better perception of life quality and lower levels of anxiety and depression. This correlation was maintained both at baseline and in the follow-up. It also showed no variability in individual scores at either follow-up, thus confirming it to be an individual characteristic, independent of external factors. An early definition of the resilience level of each patient before implantation of a defibrillator could be a useful tool in predicting the most vulnerable subjects and therefore guaranteeing the possibility of greater attention to their psychological well-being and guarantee them a targeted psychological support path.

Although it is not possible to define a statistically significant correlation between baseline levels of resilience and the onset of major cardiac events, due to the limited number of cardiac events that occurred in the follow-up period, the trend followed by our data encourages us to continue collecting data on this topic. Furthermore, given the speed and simplicity of use of the RS-14 scale, it seems promising to also further investigate the real clinical usefulness of this instrument in the cardiology field. The main limitation of this study is the total sample size. A further limitation is represented by the recruitment during the cardiological visit coinciding with the implantation of the ICD; this situation could in fact affect the patient's emotional state and represent a confounding factor in the responses to the questionnaires. Another limit of the study could be represented by the lack of collection of information regarding coping strategies, personality traits, and cognitive styles of the patients enrolled. The strengths of this study are represented by the perspective-longitudinal structure and by the double approach, intra-individual and between groups. This allowed an optimal evaluation over time of the psychophysical state of the patients. The sample examined also belongs to the real-world and is strongly representative of the population receiving a defibrillator implant. The literature shows little data in this field and our work appears innovative and a source of desirable future insights. This work confirms the need for a more careful assessment of the psychological needs and expectations of patients who undergo an ICD implant, especially in the presence of organic complications; it would be desirable to carry out a screening before implantation to identify the most vulnerable patients and direct them to a targeted path that may include professionals dedicated to the treatment of psychological complications as well as physical ones. Given the speed and simplicity of use of the RS-14 scale, it seems promising to further investigate the real clinical usefulness of this instrument in the cardiology field.

## Data Availability Statement

The original contributions presented in the study are included in the article/supplementary material, further inquiries can be directed to the corresponding author/s.

## Ethics Statement

The studies involving human participants were reviewed and approved by Ethics Committee of ASST-Settelaghi, Varese. The patients/participants provided their written informed consent to participate in this study.

## Author Contributions

CC, BC, and RD contributed to conception and design of the study. CI, CD, and FC organized the database. CI and AG performed the statistical analysis. GL and AG wrote the first draft of the manuscript. FC, MI, IC, and CD wrote sections of the manuscript. All authors contributed to the article and approved the submitted version.

## Conflict of Interest

The authors declare that the research was conducted in the absence of any commercial or financial relationships that could be construed as a potential conflict of interest.

## Publisher's Note

All claims expressed in this article are solely those of the authors and do not necessarily represent those of their affiliated organizations, or those of the publisher, the editors and the reviewers. Any product that may be evaluated in this article, or claim that may be made by its manufacturer, is not guaranteed or endorsed by the publisher.
